# Sensitive Immunoassay Detection of *Plasmodium* Lactate Dehydrogenase by Inductively Coupled Plasma Mass Spectrometry

**DOI:** 10.3389/fcimb.2020.620419

**Published:** 2021-01-11

**Authors:** Jianbing Mu, Lee L. Yu, Thomas E. Wellems

**Affiliations:** ^1^ Laboratory of Malaria and Vector Research, National Institute of Allergy and Infectious Diseases, National Institutes of Health, Rockville, MD, United States; ^2^ Chemical Sciences Division, Material Measurement Laboratory, National Institute of Standards and Technology, Gaithersburg, MD, United States

**Keywords:** Gold nanoparticles, malaria, diagnostic tests, antigen-based detection, ICP-MS

## Abstract

Rapid, reliable, and sensitive detection of *Plasmodium* infection is central to malaria control and elimination. Many Malaria Rapid Diagnostic Tests (RDTs) developed for this purpose depend upon immunoassays that can be improved by advances in bound antibody sensor technology. In a previous study, immuno-polymerase chain reaction (PCR) was shown to provide highly sensitive detection of *Plasmodium falciparum* lactate dehydrogenase (PfLDH) in monoclonal antibody (mAb) sandwich assays. Here, we show comparably high immunoassay sensitivity by inductively coupled plasma mass spectrometry (ICP-MS) detection of gold nanoparticles (AuNPs). Following capture of PfLDH with the primary mAb and binding of the AuNP-labeled detection mAb, ICP-MS signals from the AuNPs provided quantitative measures of recombinant PfLDH test dilutions and *P. falciparum-*infected erythrocytes. A detection limit of 1.5 pg/mL was achieved with the PfLDH protein. Parasitemia in cultures of *P. falciparum*-infected erythrocytes could be detected to a lower limit of 1.6 parasite/μl (p/μl) for early ring-stage forms and 0.3 p/μl for mixed stages including mature trophozoites and schizont-stages. These results show that ICP-MS detection of AuNPs can support highly sensitive and accurate detection of *Plasmodium* infection.

## Introduction

Asymptomatic and submicroscopic *Plasmodium* infections that go undetected in endemic populations and sustain transmission through mosquitoes are a continuing impediment to malaria elimination (Wu et al., 2015). Rapid and reliable diagnostic tests are needed that can detect these infections and be widely deployed for use at the primary healthcare level (Gitta and Kilian, 2020). Microscopy of Giemsa-stained blood smears falls short of these needs, not only because of the expertise and time it demands, but also by its detection threshold, estimated to be 50 to 500 parasites/μl (p/μl) under field conditions (Poschl et al., 2010). Highly sensitive molecular tests based on polymerase chain reaction (PCR) (Poschl et al., 2010), real-time quantitative PCR (qPCR) (Grignard et al., 2020), reverse transcription polymerase chain reaction (RT-PCR) (Bourgeois et al., 2010), loop-mediated isothermal amplification (LAMP) (Han et al., 2007; Kongkasuriyachai et al., 2017; Mohon et al., 2019) and a clustered, regularly-interspaced palindromic repeat (CRISPR)-based assay (Cunningham et al., 2020) are much more sensitive, detecting parasitemia as low as 2 to 5 p/μl. However, the requirements of nucleic acid extraction, amplification, and detection often limit the use of these methods in resource-poor endemic settings.

Between the relatively low sensitivity of microscopy and the high sensitivity of nucleotide sequence detection methods, a mid-range of sensitivity is offered by Rapid Diagnostic Tests (RDTs), of which some can detect as few as 10 p/μl (Hemben et al., 2018). Overall, an estimated 1.92 billion RDTs were distributed between 2010 and 2017 (Slater et al., 2015; Wu et al., 2015). Antigens detected by these RDTs include the *P. falciparum* histidine-rich protein II and III (PfHRP-II, PfHRP-III), lactate dehydrogenase of *P. falciparum* (PfLDH) as well as other *Plasmodium* species, or a *Plasmodium* aldolase, all of which are abundantly produced by the parasites during the erythrocytic cycle. Although the tests can provide rapid results and require no specific training or equipment, the performances of RDTs call for improvements on their mid-range sensitivity (Das et al., 2018) and false-negative results, which can result from loss of PfHRP-II/III expression from some *P. falciparum* strains (Gatton et al., 2017; Verma et al., 2018; Bosco et al., 2020) or bloodstream parasitemias below the detection threshold (Bell et al., 2006).

To improve the performance of RDTs, research efforts in recent years have included searches for alternative parasite antigens (Mu et al., 2017), new detection methods for hemozoin from parasite digestion of hemoglobin (Peng et al., 2014; Rifaie-Graham et al., 2019), and improved biosensors and immunosensors (Krampa et al., 2020). Electrochemical techniques, with labeled amperometric as well as label-free impedimetric strategies, have been applied to malaria diagnostic studies and outperformed optical methods (Sharma et al., 2011; Low et al., 2019; Obisesan et al., 2019). A sensitive, magnetic nanoparticle (MNP) labeled immunosensor has been shown to detect PfHRP-II with a limit of detection (LOD) of 360 pg/mL (Castilho Mde et al., 2011). Aptamer-based sensors targeting PfLDH demonstrated high sensitivity and specificity with LOD measuring 120.1 fmol/L (4 pg/ml) (Lee et al., 2012). Dirkzwager et al. (Dirkzwager et al., 2016) used aptamer functionalized microbeads to measure the enzymatic activity of recombinant PfLDH and showed a LOD of 4.9 ng/ml. Further, a portable microfluidic biosensor, integrated with the aptamer-tethered enzyme capture (APTEC) assay, was developed for highly specific detection of *P. falciparum* in cultures and clinical samples (Fraser et al., 2018). Receptor-target binding can be detected in label-free formats by piezoelectric immunosensors (Sharma et al., 2011) or indicator displacement assays (Chakma et al., 2016). Although these methods are considered to have advantages for field applications, their sensitivity (12.0 ng/ml) needs to be improved further.

Gold nanoparticles (AuNPs) offer highly useful physicochemical properties including high density, good biocompatibility, stability, and catalysis activities, which have spurred their application in bioassays and diagnostics, particularly to meet the needs of points of care (Cordeiro et al., 2016). Indeed, for malaria parasite detection, assays with AuNPs have been developed for rapid, simple, and cost-effective detection of *P. falciparum* infections, although the estimated LOD for these assays was poor (2.4 μg/ml for PfHsp70 and PfHSP90; ≈1,000 p/μl for PfLDH) (Castilho Mde et al., 2011; Guirgis et al., 2012; Jeon et al., 2013). To improve on the detection sensitivity of biosensors for AuNPs, non-optical bioassays have been developed with piezoelectric and electrochemical biosensors (Jiang et al., 2018). AuNP-labeled sandwich assay on the surface of a screen-printed gold electrode provided improved sensitivity of PfHRP2 detection to 36 pg/ml (Hemben et al., 2017) and of PfLDH to 19.0 pg/ml (Hemben et al., 2018). AuNPs have served for an electrochemical sensor in the detection of malaria parasites in clinical samples (Obisesan et al., 2019).

Inductively coupled plasma mass spectrometry (ICP-MS), is a highly sensitive technique for element and isotope analysis. The high-temperature ionization characteristics of the inductively coupled plasma (ICP), together with the sensitive and fast scanning modern mass analyzer, have boosted the capability of ICP-MS detection for biomolecules with metallic nanoparticle (NP) labels. In biomedical diagnostics, ICP-MS measurement of AuNP labels for HIV-1 p24 antigen achieved a detection limit of 1.49 pg/ml (He Q. et al., 2014). Detection of AuNPs has also served as a proxy for assays of anti-erythropoietin antibodies (Lu et al., 2009), virus-specific RNA (Hsu et al., 2011), human vascular endothelial growth factor (Thompson et al., 2010), and sialic acids on cancer cells (Zhang et al., 2016). Use of AuNPs for PfHRP-II biomolecular analysis has also been described (Wilschefski and Baxter, 2019). Because of its unmatched sensitivity for a wide range of metals and several non-metals, ICP-MS has increasingly been used for bioanalytic quantifications with element-tagged immunoassays in clinical diagnosis and single-cell analysis (Liu et al., 2014; Wilschefski and Baxter, 2019). Here, we describe the ICP-MS detection of AuNPs as a proxy for the sensitive immunoassay quantification of PfLDH, both as a purified recombinant protein and as antigen present in *P. falciparum-*infected erythrocytes.

## Methods

### *Plasmodium falciparum* Parasite Cultures and Test Sample Preparations

*P. falciparum* parasites (3D7 line) were propagated *in vitro* in O^+^ human red blood cells at 2% hematocrit in RPMI1640 medium with L-Glutamine, 25 mmol/L Hepes, 50 µg/ml Hypoxanthine (KD Medical, Columbia, MD) supplemented with 10 µg/ml gentamycin (Gibco, ThermoFisher Scientific, Grand Island, NY) and 0.5% Albumax I (ThermoFisher Scientific, Grand Island, NY) (Cranmer et al., 1997). Synchronization of parasite cultures with 5% sorbitol (Sigma) was performed as previously described (Lambros and Vanderberg, 1979). Parasitemias were determined from Giemsa-stained thin blood films, and erythrocytes per unit volume were counted by hemocytometer. The total number of parasite-infected erythrocytes was calculated as: (% parasitemia/100) × (erythrocytes/ml) × (ml of culture). For sample preparations, *P. falciparum* infected erythrocytes were washed three times with PBS (10 mmol/L $\text{P}{\text{O}}_{4}^{3-}$, 137 mmol/L NaCl, 2.7 mmol/L KCl) and lysed in a radioimmunoprecipitation assay buffer (RIPA buffer, Thermo Fisher Scientific, CA, USA) containing 1× protease inhibitor (Thermo Scientific Halt Protease Inhibitor Cocktail, Thermo Fisher Scientific, CA, USA). The samples were assayed immediately or aliquoted and stored at −20°C.

### AuNP-Labeled PfLDH Antibodies and Sample Preparations

Malaria PfLDH mouse monoclonal antibodies (mAbs) MBS498007 and MBS498008 were purchased from MyBioSource, Inc. (San Diego, CA). Detection mAb MBS498008 was labeled with AuNPs using a 60 nm gold conjugation kit (ab188216, Abcam, CA), which covalently attached the ultra-stable AuNPs. Briefly, stock mAb MBS498008 (5 mg/ml) was diluted to 0.1 mg/ml with the gold 60 nm Antibody Diluent. For each reaction, 42 µl of the 60 nm AuNP reaction buffer was added to 12 µl of diluted antibody and mixed thoroughly by pipetting up and down at least 5 times. Forty-five µl of the mixture was then transferred to a vial of 60 nm AuNPs (provided in the Abcam kit) and mixed by gently pipetting up and down. Finally, 5 µl of quencher reagent was added and the AuNP-labeled mAb was stored at 4°C before use.

### Immunoassay Detection of PfLDH Antigens

The PfLDH immunoassay was performed as previously described (Mu et al., 2017) with some modifications. Test tubes (TBS0211, Bio-Rad) were coated with anti-PfLDH mAb MBS498007 (6 μg/ml) overnight at 4°C. After three washes with PBS plus 0.05% Tween 20, the plates were blocked with tris-buffered saline, 4% bovine serum albumin (BSA), 0.05% Tween 20 (MilliporeSigma) for 2 h. Two-fold serial dilutions of recombinant PfLDH protein (MBS5308810, MyBioSource, Inc.) in 30 μl PBS buffer or 3D7 parasite lysate in RIPA buffer were added and incubated for 2 h at 37°C. After six additional washes, 30 μl of AuNP-labeled mAb MBS498008 (200 ng/ml) was incubated with the captured antigen for 1 h at 37°C. Unlabeled mAb was removed by five washes and samples were stored dry in the test tubes at 4°C before the ICP-MS was performed.

### Inductively Coupled Plasma Mass Spectrometry

ICP-MS was performed as described previously (Assumpção et al., 2013). Briefly, a 200 µl aliquot of freshly prepared aqua regia, a mixture of one part nitric acid and three parts hydrochloric acid by volume, was deposited into the test tube. The tubes were capped, and the contents were allowed to react at room temperature of 21°C on the bench overnight. The contents in the tube were quantitatively transferred to a pre-weighed 15 ml Falcon centrifuge tube (Corning 352097, Thermo Fisher Scientific) and diluted to 5 ml with locally purified water distilled below the boiling point. The mass of the resulting sample was weighed on a Mettler Toledo (Columbus, OH) model AT261 Delta Range analytical balance by difference. Procedural blanks were prepared similarly. Samples and the blanks were analyzed using the SemiQuant mode of Agilent Technologies (Santa Clara, CA) Model 7500CS inductively coupled plasma mass spectrometer. The sample introduction system of the mass spectrometer consisted of a perfluoroalkoxy alkanes (PFA) microflow (0.1 ml/min) concentric nebulizer, a PFA Scott-type double-pass spray chamber, and a sapphire injector. The SemiQuant mode is capable of quantifying elements of the entire periodic table by using the spectral information of the elements. The instrument was calibrated using a solution containing 20 µg/kg each of 31 elements (lithium, beryllium, boron, sodium, magnesium, aluminum, calcium, scandium, vanadium, chromium, manganese, cobalt, nickel, copper, zinc, arsenic, selenium, strontium, molybdenum, silver, cadmium, antimony, barium, lanthanum, europium, holmium, ytterbium, thallium, lead, thorium, and uranium) prepared by diluting Catalog No. ICP-MSCS ICP-MS Calibration Standard (High Purity Standards, Charleston, SC) with 1.5% volume fraction nitric acid (Optima grade, Thermo Fisher Scientific) in water. National Institute of Standards and Technology Standard Reference Material (SRM) 1643f, Trace Elements in Water, was measured with the samples, serving as the quality assurance for the measurement.

### Determinations of PfLDH-Based Immunoassay Sensitivity Limits

PfLDH determinations of LOD were calculated from serially-diluted samples of recombinant PfLDH protein (10 pg/ml to 0 pg/ml) by the formula: LOD = 3.3(Sy/S), where Sy and S represent the standard deviation of the response (Sy), and slope of the calibration curve (S), respectively (Evard et al., 2016). S and Sy values were obtained using “SLOPE” and “STEYX” functions in MS Excel. *P. falciparum* erythrocytic stage detection sensitivities were determined by two-fold serial-dilutions of parasite-infected cultures from 40 p/µl to 0.07 p/µl. Synchronized ring-stage and mixed stage parasite cultures were evaluated separately. ICP-MS background signals from uninfected control erythrocyte dilutions (0 p/µl) were subtracted against signals from the parasite-infected cell dilutions for analysis. One-way ANOVA and 95% confidence intervals were calculated in Prism 8 (GraphPad Software, La Jolla, CA, USA). All experiments were performed in duplicate.

## Results

### Sensitive Immunoassay Detection of PfLDH by Inductively Coupled Plasma Mass Spectrometry of Bound AuNP-Labeled Antibody

**Figure 1:**
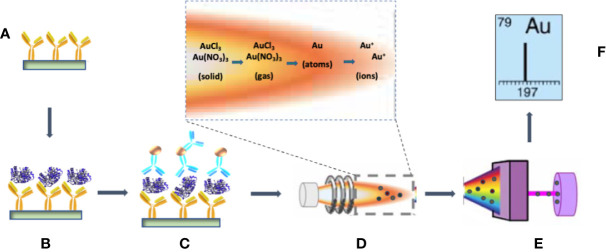
Schematic of the processes involved in the immunoassay detection of PfLDH by ICP-MS. **(A)** The test tubes are coated with the primary anti-PfLDH monoclonal antibody (mAb). **(B)** After blocking with BSA and washing, PfLDH antigens are captured by the primary mAb. **(C)** AuNP-labeled detection mAbs are bound to the capture PfLDH molecules. **(D)** The AuNP–protein complexes are dissolved in the aqua regia for injection into the spray chamber. ICP, which is a plasma sustained by inductively coupled radio frequency energy *via* a load coil after generation from a stream of argon passed through a quartz torch, serves as the ionization source. In the vaporization and ionization phase, the droplets of the sample travel through the different heating zones, in which they are dried by the plasma torch, vaporized, atomized, and finally ionized. **(E)** Ions are separated by the mass analyzer. **(F)** The detector displays the isotopic fingerprint and the analyte ion count rate, which is proportional to the mass fraction of the analyte in the sample droplets.

**Figure 2:**
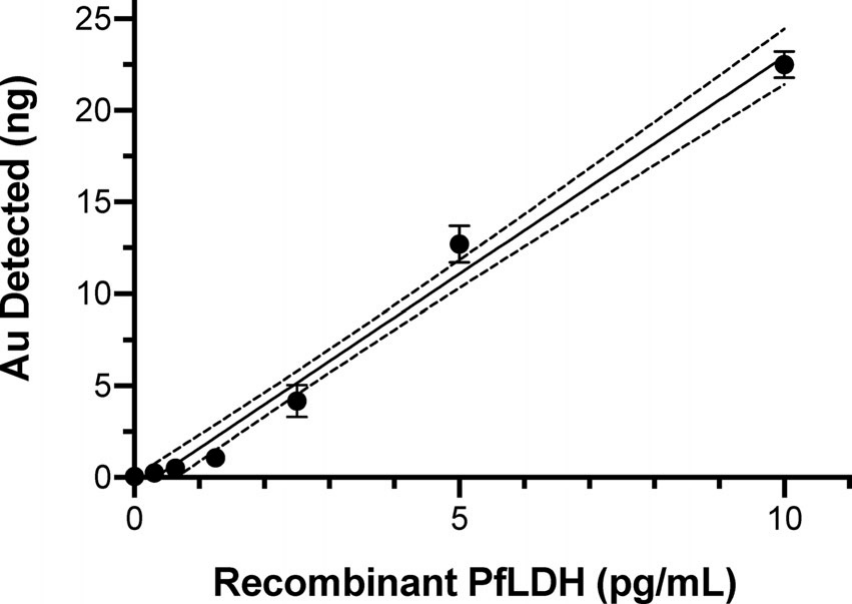
ICP-MS immunoassay results showing the elemental gold signal levels from known amounts of recombinant PfLDH protein. PfLDH was captured by the primary mAb from 30 µl volumes of the indicated concentrations of PfLDH. After binding of AuNP-labeled detection mAb and analysis by ICP-MS, the elemental gold levels were used to calculate the linear best-fit calibration line (solid line) and 95% confidence intervals (dotted lines). Average values with standard errors are shown. All experiments were performed in duplicate.

For the immunoassay detection of PfLDH, capture of the protein by the primary capture mAb and binding of the detection mAb were as previously described for high sensitivity immuno-PCR assays (Mu et al., 2017). However, instead of the biotinylated DNA tag used for immuno-PCR, the detection mAb was labeled with AuNPs (**Figure 1**). Following disruption of the immune complex with aqua regia solution, then vaporization and ionization in the nebulizer and spray chamber, gold atoms were separated according to their mass-charge ration (m/z) and quantified by ICP-MS. The amount of PfLDH in the sample is calculated with the following equation:

(1) $$C_{PfLDH\_sample}=\frac{I_{Au\_sample*}C_{PfLDH\_standard}}{I_{Au\_PfLDH\_standard}}$$

where C_PfLDH_sample_ and C_fLDH_standard_ are the concentration of PfLDH in the sample and the standard, respectively; I_Au_sample_ and I_Au_PfLDH_standard_ are the instrument response to ^197^Au in the PfLDH sample and the standard, respectively. To calibrate detection sensitivity, 30 µl volumes of a dilution series of recombinant PfLDH protein dilutions from 0.3 pg/ml to 10 pg/ml were tested by the assay protocol. ICP-MS quantification of the bound gold atoms (**Figure 2**) demonstrated sensitive detection of PfLDH protein in the pg/ml range. Note that each point on the curve was obtained from a PfLDH sample subjected to the AuNP tagged immunoassay procedure. The results from the negative controls (**Table 1**) showed that the carry-over of AuNP or unbound AuNP reagents had been effectively minimized by the multiple washes with PBS. Linear fit of the data yielded a calibration curve expressed as *y* = 2.370*x* − 0.772, *R^2^* = 0.9839, with a PfLDH LOD of 1.5 pg/ml.

**Table 1:** AuNP quantifications of parasite-infected relative to control erythrocyte samples by ICP-MS immunoassays.^a^

	Sample (p/μl)	AuNP quantification (ng)^c^	PfLDH amount (pg/ml)	PfLDH Mean Difference - Parasites *vs* control (pg/ml)	95% CI ^b^(pg/ml)	Adjusted P Value	Significance
Mixed stage parasitemia	0.07	0.60	0.58	0.56	−0.11 to 1.23	0.671	ns
	0.32	2.21	1.26	1.24	0.56 to 1.91	0.0133	*
	1.6	3.31	1.92	1.70	0.78 to 2.13	0.0046	**
	8	4.72	2.32	2.30	1.62 to 2.97	0.0002	***
	40	13.3	5.92	5.90	5.22 to 6.57	<0.0001	****
Control	0	0.05					
Ring stage parasitemia	0.07	0.57	0.57	0.55	−0.51 to 1.60	0.9562	ns
	0.32	1.30	0.87	0.85	−0.20 to 1.90	0.4836	ns
	1.6	2.73	1.48	1.46	0.65 to 2.75	0.0101	*
	8	4.97	2.42	2.40	1.36 to 3.45	0.0004	***
	40	9.56	4.36	4.34	3.29 to 5.39	<0.0001	****
Control	0	0.05					

a One-way ANOVA analysis was used for multiple comparisons test.

b 95% confidence interval of difference. Significance indications: ns, not significant; *P < 0.05; **P < 0.01; ***P < 0.001; ****P < 0.0001.

c Mean of two independent biological repeats.

### Sensitive Detection of *Plasmodium falciparum*-Infected Erythrocytes by the Inductively Coupled Plasma Mass Spectrometry PfLDH Immunoassay

**Figure 3:**
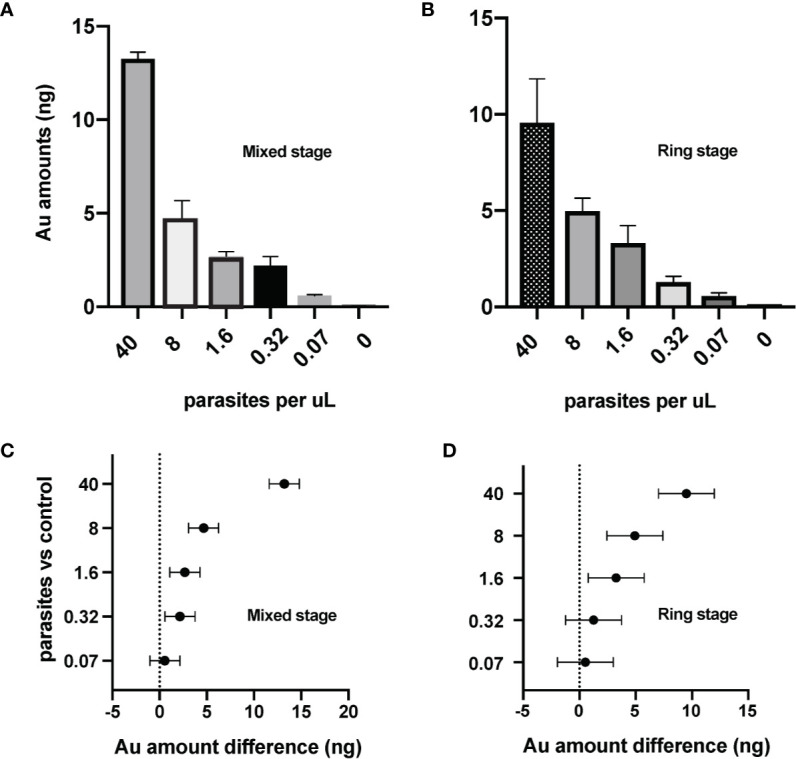
Sensitive detection of the *P. falciparum* parasites by ICP-MS immunoassay targeting the PfLDH antigen. The ICP-MS method is able to detect AuNPs from different samples of **(A)** mixed stage and **(B)** ring stage *P. falciparum*-infected erythrocytes. Limits of detection (LOD) of the ICP-MS PfLDH immunoassay are estimated to be **(C)** 0.3 p/µl for the mixed stages, and **(D)** 1.6 p/µl for the ring stages. The horizontal bars represent the 95% confidence interval. The averages are from experiments repeated separately on different days.

We next evaluated the sensitivity of the ICP-MS PfLDH immunoassay for detection of *P.* *falciparum*-infected erythrocytes. Experiments were performed with mixed stage-infected erythrocytes (**Figure 3A**) or with synchronized ring stage-infected erythrocytes (**Figure 3B**). The Au signals at 0.57 to 0.60 ng from the lowest test parasitemia (0.07 p/µl) were more than 10× higher compared to that of the control sample (0.05 ng, **Table 1**), testifying to the high sensitivity of ICP-MS based immunoassay in detection of *P. falciparum* infections. Statistical analysis with 95% confidence interval of difference (**Table 1**), showed that the significant difference between the test samples and control could be confidently detected down to a level of 0.32 p/µl for mixed stage (**Figure 3C**) or 1.6 p/µl for ring stage test samples (**Figure 3D**).

## Discussion

Recent years have seen remarkable advancement in the application of ICP-MS technology for molecular diagnostics, particularly when it is applied to molecular targets with metal nanoparticle labels (Wilschefski and Baxter, 2019). Adding to the growing list, the ICP-MS PfLDH immunoassay described here detects *P. falciparum*-infected erythrocytes at the level of ≈1 p/µl, comparable to the detection limit of immuno-PCR in sandwich assays with the same mAbs (Mu et al., 2017).

A mass spectrometer is an ion counter. By labeling PfLDH with AuNP, the analyte density as defined by the number of PfLDH molecules is effectively multiplied by a factor which is the number of Au atoms in the average number of AuNPs attached to each PfLDH molecule. Note that the number of Au atoms, and hence the signal enhancement factor by ICP-MS detection, is proportional to the third power of the diameter of attached AuNPs. It may therefore be possible to improve detection sensitivity further by using AuNPs of larger diameter for labeling. With the 60 nm AuNP, ICP-MS is already capable of detecting the amount of Au at 10× below the amount associated with 0.07 p/µl, which is far below the detection limit of 0.32 p/µl to 1.6 p/µl discussed above. Therefore, the limiting factor to the LOD of the parasite is the reproducibility of the sandwich immunoassay procedure especially at low PfLDH concentration end rather than ICP-MS detection of Au. Strategies such as chemically modified nucleotide-based elemental tags (Hu et al., 2019), three-dimensional structure modifications of the AuNPs (Wang et al., 2018), rolling circle amplification of parasite DNA detected with AuNP-sensors (He Y. et al., 2014), and multiplex assays of biomarker proteins with AuNP-tagged antibodies (Terenghi et al., 2009) may also provide opportunities for higher sensitivity detection by ICP-MS. The ultimate goal will be to develop high-sensitivity detection of AuNPs with practical, field-robust devices at point of care. Design and development of the necessary equipment, the possibility of which brings to mind the successful production of a low-cost, portable, field-programmable nuclear magnetic resonance spectrometer (Peng et al., 2014), will support this goal.

## Data Availability Statement

The raw data supporting the conclusions of this article will be made available by the authors, without undue reservation.

## Author Contributions

JM and TW initiated the study and drafted the manuscript. JM and LY conducted the lab work and analyzed the data. All authors contributed to the article and approved the submitted version.

## Funding

This work was supported by the Intramural Research Program of the Division of Intramural Research, National Institute of Allergy and Infectious Diseases, National Institutes of Health.

## Disclaimer

Certain commercial equipment, instruments or materials are identified in this manuscript to adequately specify the experimental procedure. Such identification does not imply recommendation or endorsement by the National Institute of Standards and Technology, nor does it imply that the materials or equipment identified are necessarily the best available for the purpose.

## Conflict of Interest

The authors declare that the research was conducted in the absence of any commercial or financial relationships that could be construed as a potential conflict of interest.

## References

[B1] Assumpção T. C. Ma D. Schwarz A. Reiter K. Santana J. M. Andersen J. F. (2013). Salivary antigen-5/CAP family members are Cu2+-dependent antioxidant enzymes that scavenge O_2-_. and inhibit collagen-induced platelet aggregation and neutrophil oxidative burst. J. Biol. Chem. 288, 14341–14361. 10.1074/jbc.M113.466995 23564450 PMC3656290

[B2] Bell D. Wongsrichanalai C. Barnwell J. W. (2006). Ensuring quality and access for malaria diagnosis: how can it be achieved? Nat. Rev. Microbiol. 4, S7–20. 10.1038/nrmicro1525 16912713

[B3] Bosco A. B. Anderson K. Gresty K. Prosser C. Smith D. Nankabirwa J. I. (2020). Molecular surveillance reveals the presence of pfhrp2 and pfhrp3 gene deletions in Plasmodium falciparum parasite populations in Uganda 2017-2019. Malar J. 19, 300. 10.1186/s12936-020-03362-x 32843041 PMC7449024

[B4] Bourgeois N. Boutet A. Bousquet P. J. Basset D. Douard-Enault C. Charachon S. (2010). Comparison of three real-time PCR methods with blood smears and rapid diagnostic test in Plasmodium sp. infection. Clin. Microbiol. Infect. 16, 1305–1311. 10.1111/j.1469-0691.2009.02933.x 19840032

[B5] Castilho Mde S. Laube T. Yamanaka H. Alegret S. Pividori M. I. (2011). Magneto immunoassays for Plasmodium falciparum histidine-rich protein 2 related to malaria based on magnetic nanoparticles. Anal. Chem. 83, 5570–5577. 10.1021/ac200573s 21619038

[B6] Chakma B. Jain P. Singh N. K. Goswami P. (2016). Development of an indicator displacement based detection of malaria targeting HRP-II as biomarker for application in point-of-care settings. Anal. Chem. 88, 10316–10321. 10.1021/acs.analchem.6b03315 27659695

[B7] Cordeiro M. Ferreira Carlos F. Pedrosa P. Lopez A. Baptista P. V. (2016). Gold nanoparticles for diagnostics: advances towards points of care. Diagnostics (Basel) 6, 43. 10.3390/diagnostics6040043 PMC5192518 27879660

[B8] Cranmer S. L. Magowan C. Liang J. Coppel R. L. Cooke B. M. (1997). An alternative to serum for cultivation of Plasmodium falciparum in vitro. Trans. R Soc. Trop. Med. Hyg. 91, 363–365. 10.1016/S0035-9203(97)90110-3 9231219

[B9] Cunningham C. H. Hennelly C. M. Lin J. T. Ubalee R. Boyce R. M. Mulogo E. M. (2020). A novel CRISPR-based malaria diagnostic capable of Plasmodium detection, speciation, and drug-resistance genotyping. bioRxiv. 2020.2004.2001.017962. 10.1101/2020.04.01.017962 PMC8213918 34139428

[B10] Das S. Peck R. B. Barney R. Jang I. K. Kahn M. Zhu M. (2018). Performance of an ultra-sensitive Plasmodium falciparum HRP2-based rapid diagnostic test with recombinant HRP2, culture parasites, and archived whole blood samples. Malar J. 17, 118. 10.1186/s12936-018-2268-7 29549888 PMC5857316

[B11] Dirkzwager R. M. Liang S. Tanner J. A. (2016). Development of aptamer-based point-of-care diagnostic devices for malaria using three-dimensional printing rapid prototyping. ACS Sensors 1, 420–426. 10.1021/acssensors.5b00175

[B12] Evard H. Kruve A. Leito I. (2016). Tutorial on estimating the limit of detection using LC-MS analysis, part II: Practical aspects. Anal. Chim. Acta 942, 40–49. 10.1016/j.aca.2016.08.042 27720120

[B13] Fraser L. A. Kinghorn A. B. Dirkzwager R. M. Liang S. Cheung Y. W. Lim B. (2018). A portable microfluidic Aptamer-Tethered Enzyme Capture (APTEC) biosensor for malaria diagnosis. Biosens. Bioelectron 100, 591–596. 10.1016/j.bios.2017.10.001 29032164

[B14] Gatton M. L. Dunn J. Chaudhry A. Ciketic S. Cunningham J. Cheng Q. (2017). Implications of parasites lacking Plasmodium falciparum histidine-rich protein 2 on malaria morbidity and control when rapid diagnostic tests are used for diagnosis. J. Infect. Dis. 215, 1156–1166. 10.1093/infdis/jix094 28329034

[B15] Gitta B. Kilian N. (2020). Diagnosis of malaria parasites plasmodium spp. in endemic areas: current strategies for an ancient disease. Bioessays 42, e1900138. 10.1002/bies.201900138 31830324

[B16] Grignard L. Nolder D. Sepulveda N. Berhane A. Mihreteab S. Kaaya R. (2020). A novel multiplex qPCR assay for detection of Plasmodium falciparum with histidine-rich protein 2 and 3 (pfhrp2 and pfhrp3) deletions in polyclonal infections. EBioMedicine 55, 102757. 10.1016/j.ebiom.2020.102757 32403083 PMC7218259

[B17] Guirgis B. S. Sa E Cunha C. Gomes I. Cavadas M. Silva I. Doria G. (2012). Gold nanoparticle-based fluorescence immunoassay for malaria antigen detection. Anal. Bioanal. Chem. 402, 1019–1027. 10.1007/s00216-011-5489-y 22089818

[B18] Han E. T. Watanabe R. Sattabongkot J. Khuntirat B. Sirichaisinthop J. Iriko H. (2007). Detection of four Plasmodium species by genus- and species-specific loop-mediated isothermal amplification for clinical diagnosis. J. Clin. Microbiol. 45, 2521–2528. 10.1128/JCM.02117-06 17567794 PMC1951264

[B19] He Q. Zhu Z. L. Jin L. L. Peng L. Guo W. Hu S. H. (2014). Detection of HIV-1 p24 antigen using streptavidin-biotin and gold nanoparticles based immunoassay by inductively coupled plasma mass spectrometry. J. Anal. Atomic Spectrom. 29, 1477–1482. 10.1039/C4JA00026A

[B20] He Y. Chen D. Li M. Fang L. Yang W. Xu L. (2014). Rolling circle amplification combined with gold nanoparticles-tag for ultra sensitive and specific quantification of DNA by inductively coupled plasma mass spectrometry. Biosens. Bioelectron 58, 209–213. 10.1016/j.bios.2014.02.072 24637171

[B21] Hemben A. Ashley J. Tothill I. E. (2017). Development of an immunosensor for PfHRP 2 as a biomarker for malaria detection. Biosens. (Basel) 7, 28. 10.3390/bios7030028 PMC5618034 28718841

[B22] Hemben A. Ashley J. Tothill I. E. (2018). An immunosensor for parasite lactate dehydrogenase detection as a malaria biomarker - comparison with commercial test kit. Talanta 187, 321–329. 10.1016/j.talanta.2018.04.086 29853054

[B23] Hsu I. H. Chen W. H. Wu T. K. Sun Y. C. (2011). Gold nanoparticle-based inductively coupled plasma mass spectrometry amplification and magnetic separation for the sensitive detection of a virus-specific RNA sequence. J. Chromatogr. A 1218, 1795–1801. 10.1016/j.chroma.2011.02.005 21376334

[B24] Hu Z. Sun G. Jiang W. Xu F. Zhang Y. Xia M. (2019). Chemical-modified nucleotide-based elemental tags for high-sensitive immunoassay. Anal. Chem. 91, 5980–5986. 10.1021/acs.analchem.9b00405 30973226

[B25] Jeon W. Lee S. Manjunatha D. H. Ban C. (2013). A colorimetric aptasensor for the diagnosis of malaria based on cationic polymers and gold nanoparticles. Anal. Biochem. 439, 11–16. 10.1016/j.ab.2013.03.032 23583275

[B26] Jiang P. Wang Y. Zhao L. Ji C. Chen D. Nie L. (2018). Applications of gold nanoparticles in non-optical biosensors. Nanomaterials (Basel) 8, 977. 10.3390/nano8120977 PMC6315477 30486293

[B27] Kongkasuriyachai D. Yongkiettrakul S. Kiatpathomchai W. Arunrut N. (2017). Loop-mediated isothermal amplification and LFD combination for detection of Plasmodium falciparum and Plasmodium vivax. Methods Mol. Biol. 1572, 431–443. 10.1007/978-1-4939-6911-1_28 28299704

[B28] Krampa F. D. Aniweh Y. Kanyong P. Awandare G. A. (2020). Recent advances in the development of biosensors for malaria diagnosis. Sensors (Basel) 20, 799. 10.3390/s20030799 PMC7038750 32024098

[B29] Lambros C. Vanderberg J. P. (1979). Synchronization of Plasmodium falciparum erythrocytic stages in culture. J. Parasitol. 65, 418–420. 10.2307/3280287 383936

[B30] Lee S. Song K. M. Jeon W. Jo H. Shim Y. B. Ban C. (2012). A highly sensitive aptasensor towards Plasmodium lactate dehydrogenase for the diagnosis of malaria. Biosens. Bioelectron 35, 291–296. 10.1016/j.bios.2012.03.003 22459583

[B31] Liu R. Wu P. Yang L. Hou X. Lv Y. (2014). Inductively coupled plasma mass spectrometry-based immunoassay: a review. Mass Spectrom. Rev. 33, 373–393. 10.1002/mas.21391 24272753

[B32] Low Y. K. Chan J. Soraya G. V. Buffet C. Abeyrathne C. D. Huynh D. H. (2019). Development of an ultrasensitive impedimetric immunosensor platform for detection of Plasmodium lactate dehydrogenase. Sensors (Basel) 19, 2446. 10.3390/s19112446 PMC6603725 31146340

[B33] Lu Y. Wang W. Xing Z. Wang S. Cao P. Zhang S. (2009). Development of an ICP-MS immunoassay for the detection of anti-erythropoietin antibodies. Talanta 78, 869–873. 10.1016/j.talanta.2008.12.065 19269443

[B34] Mohon A. N. Getie S. Jahan N. Alam M. S. Pillai D. R. (2019). Ultrasensitive loop mediated isothermal amplification (US-LAMP) to detect malaria for elimination. Malar J. 18, 350. 10.1186/s12936-019-2979-4 31619258 PMC6796404

[B35] Mu J. Andersen J. F. Valenzuela J. G. Wellems T. E. (2017). High-sensitivity assays for Plasmodium falciparum infection by immuno-polymerase chain reaction detection of PfIDEh and PfLDH antigens. J. Infect. Dis. 216, 713–722. 10.1093/infdis/jix369 28934434 PMC5854022

[B36] Obisesan O. R. Adekunle A. S. Oyekunle J. Sabu T. Nkambule T. T. I. Mamba B. B. (2019). Development of electrochemical nanosensor for the detection of malaria parasite in clinical samples. Front. Chem. 7, 89. 10.3389/fchem.2019.00089 30859097 PMC6397833

[B37] Peng W. K. Kong T. F. Ng C. S. Chen L. Huang Y. Bhagat A. A. (2014). Micromagnetic resonance relaxometry for rapid label-free malaria diagnosis. Nat. Med. 20, 1069–1073. 10.1038/nm.3622 25173428

[B38] Poschl B. Waneesorn J. Thekisoe O. Chutipongvivate S. Karanis P. (2010). Comparative diagnosis of malaria infections by microscopy, nested PCR, and LAMP in northern Thailand. Am. J. Trop. Med. Hyg. 83, 56–60. 10.4269/ajtmh.2010.09-0630 20595478 PMC2912576

[B39] Rifaie-Graham O. Pollard J. Raccio S. Balog S. Rusch S. Hernandez-Castaneda M. A. (2019). Hemozoin-catalyzed precipitation polymerization as an assay for malaria diagnosis. Nat. Commun. 10, 1369. 10.1038/s41467-019-09122-z 30911004 PMC6433922

[B40] Sharma M. K. Rao V. K. Merwyn S. Agarwal G. S. Upadhyay S. Vijayaraghavan R. (2011). A novel piezoelectric immunosensor for the detection of malarial Plasmodium falciparum histidine rich protein-2 antigen. Talanta 85, 1812–1817. 10.1016/j.talanta.2011.07.008 21872024

[B41] Slater H. C. Ross A. Ouedraogo A. L. White L. J. Nguon C. Walker P. G. (2015). Assessing the impact of next-generation rapid diagnostic tests on Plasmodium falciparum malaria elimination strategies. Nature 528, S94–101. 10.1038/nature16040 26633771

[B42] Terenghi M. Elviri L. Careri M. Mangia A. Lobinski R. (2009). Multiplexed determination of protein biomarkers using metal-tagged antibodies and size exclusion chromatography–inductively coupled plasma mass spectrometry. Anal. Chem. 81, 9440–9448. 10.1021/ac901853g 19908906

[B43] Thompson D. F. Eborall W. Dinsmore A. Smith C. J. Duckett C. J. (2010). Development and validation of a NANOGold immunoassay for the detection of vascular endothelial growth factor (VEGF) in human serum using inductively coupled plasma mass spectrometry. Rapid Commun. Mass Spectrom. 24, 927–932. 10.1002/rcm.4456 20196195

[B44] Verma A. K. Bharti P. K. Das A. (2018). HRP-2 deletion: a hole in the ship of malaria elimination. Lancet Infect. Dis. 18, 826–827. 10.1016/S1473-3099(18)30420-1 30064667

[B45] Wang X. Du D. Dong H. Song S. Koh K. Chen H. (2018). para-Sulfonatocalix[4]arene stabilized gold nanoparticles multilayers interfaced to electrodes through host-guest interaction for sensitive ErbB2 detection. Biosens. Bioelectron 99, 375–381. 10.1016/j.bios.2017.08.011 28802750

[B46] Wilschefski S. C. Baxter M. R. (2019). Inductively coupled plasma mass spectrometry: introduction to analytical aspects. Clin. Biochem. Rev. 40, 115–133. 10.33176/AACB-19-00024 31530963 PMC6719745

[B47] Wu L. Van Den Hoogen L. L. Slater H. Walker P. G. Ghani A. C. Drakeley C. J. (2015). Comparison of diagnostics for the detection of asymptomatic Plasmodium falciparum infections to inform control and elimination strategies. Nature 528, S86–S93. 10.1038/nature16039 26633770

[B48] Zhang X. Chen B. He M. Zhang Y. Peng L. Hu B. (2016). Boronic acid recognition based-gold nanoparticle-labeling strategy for the assay of sialic acid expression on cancer cell surface by inductively coupled plasma mass spectrometry. Analyst 141, 1286–1293. 10.1039/C5AN02402A 26811850

